# Secular trends of macrosomia in southeast China, 1994-2005

**DOI:** 10.1186/1471-2458-11-818

**Published:** 2011-10-20

**Authors:** Yanyu Lu, Jun Zhang, Xinrong Lu, Wei Xi, Zhu Li

**Affiliations:** 1 School of Public Health, Peking University Health Science Center, Beijing 100191, China; 2 Capital Institute of Pediatrics, Beijing 100020, China; 3 MOE-Shanghai Key Laboratory of Children's Environmental Health, Xinhua Hospital, Shanghai Jiao Tong University School of Medicine, Shanghai 200092, China; 4 Chinese Jilin Center For Disease Control and Prevention, Changchun 130062, China; 5 School of Public Health, Tianjin Medical University, Tianjin 300100, China

## Abstract

**Background:**

The rate of macrosomia (birth weight≥4, 000 g) increased over the past four decades in many parts of the world. Macrosomia is associated not only with higher risks of maternal and neonatal complications but also with health risks in adulthood. We examined trends in neonatal macrosomia and large-for-gestational-age (LGA) births among singleton, live, term and postterm births (≥37 complete weeks' gestation) in southeast China from 1994 to 2005 and explored possible causes of the temporal trends.

**Methods:**

Data from Perinatal Health Care Surveillance System in 12 cities and counties in southeast China were analyzed for trends in birth weight, neonatal macrosomia and LGA from 1994 to 2005. A total of 594, 472 singleton live births were included. We conducted multiple logistic regression analyses to relate these trends to changes in maternal and pregnancy characteristics.

**Results:**

The rate of macrosomia rose from 6.00% in 1994 to 8.49% in 2000 and then levelled off to 7.83% in 2005. Similar trends were observed in mean birth weight. The incidence of LGA births increased continuously from 13.72% in 1994 to 18.08% in 2000, but the LGA rate remained relatively stable from 2002 to 2005. There was a decrease in gestational age and a significant increase in frequency of prelabor caesarean delivery from 1994 to 2005. In an adjusted multivariable model, the increase in LGA rate from 1994 to 2000 was associated with increasing net gestational weight gain, maternal age, maternal height and maternal education. But they didn't fully explain the increase. The trends of 2002-2005 LGA declined after adjusted for maternal and neonatal characteristics.

**Conclusions:**

In southeast China, the incidence of macrosomia increased from 1994 to 2000 was mainly related to increasing net gestational weight gain. The incidence of macrosomia has levelled off in recent years partly due to increasing use of prelabor caesarean delivery and earlier delivery and partly due to moderation of gestational weight gain.

## Background

Birth weight and rate of macrosomia and large for gestational age (LGA) increased over the past four decades in many countries [1-7]. Temporal increases in maternal body mass index (BMI), gestational weight gain, maternal height, diabetes, reduced maternal cigarette smoking and changes in sociodemographic factors have contributed to these trends [8-11].

However, a recent study showed that the rate of macrosomia was decreasing in the United States since early 1990s [12] despite the fact that the prevalence of obesity was increasing [13]. This phenomenon was attributed to the increased use of labor induction that shortened the duration of gestation and, therefore, reduced both mean birth weight and rate of macrosomia in one study [14]. But another study showed that trends in maternal and neonatal characteristics, changes in obstetric practices, and concurrent decreases in gestational length didn't explain the recent decrease in birth weight and incidence of LGA using data from the U.S. National Center for Health Statistics [15].

With rapid economic growth in China in the past three decades, investments in education, healthcare and sanitation have increased accordingly. Chinese national health services survey showed that birth weight increased from 3186 g in 1993 to 3284 g in 1998 and to 3307 g in 2003 [16]. A rapid increase in rate of macrosomia has been reported in China. For example, Liu et al. found that in a hospital in Yantai the incidence of macrosomia was 2.6%, 6.9% and 13.2% in the 1970s, 1980s and 1990s respectively. Macrosomia was associated with significant differences in the following: maternal height, weight, abdominal circumference and gestational diabetes in these three periods [17]. In Shanghai, the rates of macrosomia increased by 50% between 1989 and 1999 [18]. However, few systematic studies were performed on reasons for these trends. A Perinatal Health Care Surveillance System (PHCSS) in southeast China allowed us to study trends in macrosomia in a population-based setting from 1994 to 2005 and to identify possible risk factors.

## Methods

### Data source and study population

We used data from the population-based PHCSS that was established along with the community intervention trial for preventing neural tube defects with peri-conceptive supplementation of 400 μg folic acid daily. The intervention trial was conducted in one northern province (Hebei) and two southeast provinces (Zhejiang and Jiangsu) from October 1993 through December, 1996. The original project design and results of the neural tube defects prevention program have already been published elsewhere [19]. However, the PHCSS continued to function after the original project ended in 1996. There were 19 cities and counties in the two southeast provinces implementing the surveillance program without interruption from the start of intervention trial until 2005. The study was approved by the institutional review board (IRB) of Peking University Health Science Center.

In the PHCSS, we collected information on parental demographics, maternal medical and reproductive history, medical conditions during pregnancy, labour and delivery summary and postpartum conditions. Each woman was issued a Perinatal Health Care Booklet and was assigned a unique identification number when she was at her marital registration, at the first prenatal care visit, or when she sought perinatal care services. The women were followed during their pregnancy, delivery and the immediate postpartum period by local health care professionals. After postpartum discharge, the booklet was collected and all data recorded in the booklet were computerized by trained staff at each county using a standardized data entry application with built-in data checking function. The National Center for Maternal and Infant Health was in charge of data cleaning and editing. In 2001, a computer electronic record system was established in the southeast provinces. Information on each prenatal visit, labor and delivery summary, and postpartum visit was entered by the health care providers on site and uploaded to the county server each day. The county then sent the data electronically every month to the National Center for Maternal and Infant Health for cleaning and editing.

We selected 12 cities and counties from 19 surveillance sites because they had better data quality. They had the most complete data with, on average, over 90% of the births in the site, except for 2001. In 2001 problems with transitioning from paper to electronic registration resulted in inadequate data on large numbers infants. Thus, we dropped the 2001 data from the study. The data in this paper goes from 1994 through 2005 with the exception of 2001. The number of infants with missing birth weight was no more than 2.0% at all sites. To identify and delete implausible birthweight-gestational age combinations we used Alexander's algorithm [20]. The number of implausible birthweight-gestational age combinations was under 2.0% at all sites.

There were 692, 330 live births born from January 1, 1994 to December 31, 2005 in our study sites. We excluded 21, 914 births with missing birth weight, or gestational age values outside the range of 20-44 weeks or implausible birthweight-gestational age combinations. After exclusion of 40, 506 multiple births and preterm newborns, there were 629, 910 live-born singletons≥37 gestational weeks. Not all births were captured in 2001, we, therefore, deleted 2001 data, leaving 594, 472 births for analysis.

### Outcome and explanatory variables

Birth weight was recorded in 50 grams. Macrosomia was defined as birth weight ≥4, 000 g, irrespective of gestational age [21]. Gestational age was computed on the interval between the first day of last menstrual period and the date of birth. We defined small for gestational age (SGA) as birth weight less than 10th percentile and large for gestational age (LGA) as birth weight greater than 90th percentile at each gestational week according to the new method recently published in Lancet [22]. We calculated mean birth weight and standard deviation of birth weight at 40 weeks based on 1994 to 2005 data, not including 2001, as a reference point in order to produce the graphs related to birth weight, gestational age percentile. We examined macrosomia in the context of maternal age, education, maternal residence, maternal height, maternal early pregnancy BMI, net gestational weight gain, parity and infant gender. Maternal early pregnancy BMI was based on measured height and weight at the first prenatal visit during the first trimester. If women who didn't take a prenatal care during the first trimester, we used the height and weight measured at premarital consultation. According to WHO reference [23], maternal BMI was grouped into four categories: < 18.5 kg/m^2^(Under weight), 18.5-22.9 kg/m^2^(Normal), 23-24.9 kg/m^2^(Overweight), ≥25 kg/m^2^(Obesity). Gestational weight gain was defined as the last measured weight in the 3rd trimester minus the early pregnancy weight or weight measured at premarital consultation, referring to IOM [24]. Net gestational weight gain was calculated as gestational weight gain minus birth weight and was classified into three groups: < 6.5 kg, 6.5-12.5 kg, > 12.5 kg based on interquartile range. Maternal age was defined in completed years at delivery: 24 or less, 25-29, 30-34, or 35 or more. Maternal education was categorized as elementary school or less, junior middle school, high school, college or above.

### Data analysis

χ^2 ^tests for linear trend were used for dichotomous outcomes, and one-way analyses of variance with linear contrast were used for continuous outcomes. We used logistic regression to examine the associations between maternal and perinatal characteristics and risk of delivering macrosomia. We also estimated a series of multiple logistic regression models to examine the changes of single-year effects on LGA after sequentially adjusting for related factors. Values for maternal early pregnancy BMI and net gestational weight gain were missing for a substantial proportion of the study cohort. We assessed the impact of missing values by creating a category of "unknown" for factors with a substantial proportion of missing values or by deleting the cases with missing values. We then compared the results. The direction of the results didn't alter. Thus, the results presented here were the model without missing values. The statistical analyses were conducted with SPSS 11.5 (SPSS Inc., Chicago, IL).

## Results

**Figure 1:**
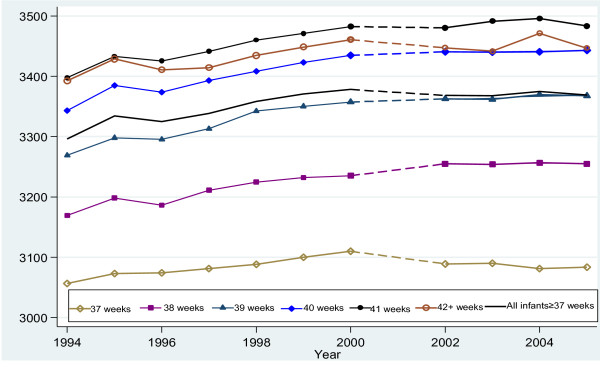
**Secular trends in mean birth weight by gestational age among singleton live births at ≥37 weeks of gestation**.

Over the 11-year period, mean birth weight for all term and postterm infants increased from 3296 g in 1994 to 3378 g in 2000, then levelled off to 3369 g in 2005. As show in Figure 1, the increments of birth weight differed considerably by gestational age. Mean birth weight rose the most at 38-41 weeks (more than 80 g) from 1994 to 2005. The incremental rise of birth weight between 2002 and 2005 was smaller than that between 1994 and 2000.

**Figure 2:**
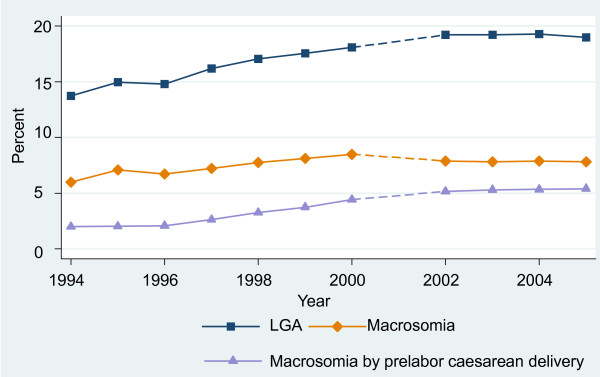
**Trends in prevalence of macrosomia, macrosomia delivered by prelabor caesarean delivery and large-for-gestational age(LGA) births from 1994 to 2005 among singleton live births≥37 weeks in southeast China**.

The trend of incidence of macrosomia followed the pattern of birth weight for all term and postterm infants. The proportion of macrosomia increased steadily from 6.00% at 1994 to 8.49% at 2000 and moderated to 7.83% at 2005 (Figure 2). In 1994, one-third of macrosomic infants were delivered by prelabor caesarean delivery rising to 69% by 2005. The incidence of LGA increased significantly from 13.72% in 1994 to 18.98% in 2005 while the percentage of SGA infants declined steadily from 11.95% in 1994 to 7.00% in 2005.

**Figure 3:**
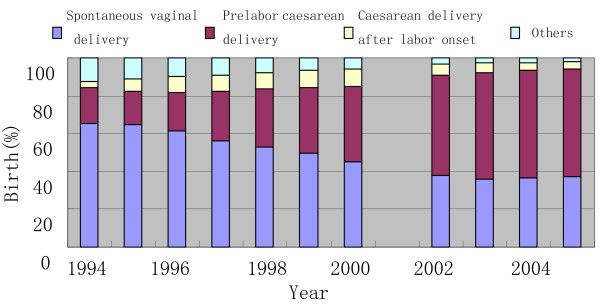
**Secular trends in delivery mode information**.

The mode of delivery changed greatly in this period at our study sites. Figure 3 shows that the rate of prelabor caesarean delivery for births at or above 37 weeks nearly tripled over the 11-year period. Between 1994 and 2000, the rate of prelabor caesarean delivery rose from 19.41% in 1994 to 39.77% in 2000. It further increased to 53.04% by 2002 and remained between 53% and 57% from 2002 through 2005. The rate of spontaneous vaginal delivery decreased from 65.50% to 37.25% during this period.

Table 1 shows the changes of maternal and infants characteristics by year of delivery. During the past 11 years, maternal height and age all increased. Maternal early pregnancy BMI increased. The proportion of underweight remained relatively stable and the proportion of normal weight declined. But the proportions of overweight and obesity all rose more quickly especially in the later years. Mean net gestational weight gain increased between 1994 and 2000, and remained stable between 2002 and 2005. The proportion of birth to primiparous women increased. Maternal education greatly improved. The percentage of women who had high school or above education increased from 16.43% to 51.16%. Urbanization of these sites accelerated. More and more women lived in city. Mean gestational age declined slightly from 280.4 days in 1994 to 279.8 days in 2000, and to 278.4 days in 2002. It remained between 278.4 days and 278.2 days from 2002 through 2005. A larger proportion of infants was born at 38 and 39 weeks while a smaller proportion was born at 41 weeks and ≥42 weeks. Due to a large sample size, all differences of these temporal trends were statistically significant at P < 0.05.

**Table 1:** Temporal trends of maternal and infants characteristics in southeast China from 1994 to 2005.

Variables	1994	1995	1996	1997	1998	1999	2000	2002	2003	2004	2005
**No. of Newborns**	64, 986	67, 353	58, 548	58, 854	53, 011	49, 102	54, 208	45, 104	42, 566	53, 103	47, 637
Male (%)*	52.03	51.58	51.67	51.71	51.67	51.60	51.80	52.54	52.54	52.81	52.30
Nulliparity (%)*	84.32	84.54	82.80	83.95	84.24	85.93	86.15	87.94	88.76	88.52	87.00
**Gestational age(d, mean ± SD)#**	280.38 ± 9.21	280.81 ± 9.10	280.63 ± 9.02	280.04 ± 8.72	280.00 ± 8.60	280.07 ± 8.42	279.82 ± 8.31	278.39 ± 8.04	278.25 ± 7.96	278.49 ± 7.86	278.15 ± 7.87
Gestational age distribution (%)*											
**37 weeks**	5.60	4.93	5.01	5.28	5.13	4.76	4.66	5.92	5.73	5.19	5.64
**38 weeks**	13.91	12.80	13.01	13.49	13.50	12.92	13.41	16.53	16.99	16.22	16.81
**39 weeks**	26.58	26.43	26.84	28.33	28.15	28.88	29.72	33.05	33.6	34.19	34.90
**40 weeks**	29.59	30.21	30.44	30.94	32.00	32.34	32.60	29.82	29.81	30.23	29.48
**41 weeks**	16.77	17.72	17.25	16.23	15.85	16.04	14.84	11.70	11.11	11.19	10.39
**≥42 weeks**	7.55	7.90	7.46	5.73	5.37	5.05	4.77	2.98	2.76	2.98	2.78
Maternal age (%)*											
**≤24 y**	58.24	54.45	50.61	46.39	44.59	46.23	47.38	50.34	49.22	46.70	44.96
**25-29 y**	30.78	34.23	36.31	40.39	42.06	41.86	39.59	36.70	37.72	40.61	41.13
**30-34 y**	10.10	10.41	12.16	12.09	11.71	10.12	11.10	10.81	10.63	10.29	10.98
**≥35 y**	0.87	0.91	0.91	1.14	1.63	1.78	1.93	2.15	2.43	2.40	2.93
Maternal education (%)*											
**College or above**	3.03	3.29	3.44	4.48	5.51	6.62	8.06	10.73	12.14	16.51	20.71
**High school**	13.40	14.46	15.38	16.96	19.08	21.99	23.71	28.83	30.99	32.96	30.45
**Junior school**	58.79	61.04	62.26	63.35	62.66	60.57	58.57	53.42	50.56	45.28	43.07
**Elementary school or less**	24.77	21.20	18.92	15.21	12.75	10.82	9.66	7.01	6.31	5.25	5.77
Maternal height(%)*											
**≤1.56 m**	27.98	26.47	26.96	26.16	26.37	25.85	25.56	25.02	24.59	23.39	23.61
**1.57-1.61 m**	44.06	44.95	44.86	45.05	44.29	44.66	44.55	44.24	44.41	44.91	45.08
**≥1.62 m**	27.95	28.58	28.18	28.79	29.34	29.48	29.89	30.74	31.00	31.69	31.31
Maternal BMI(%)*											
**< 18.5 kg/m**^**2**^	19.07	20.44	19.61	21.22	20.48	20.61	20.90	20.64	20.87	19.00	18.88
**18.5-22.9 kg/m**^**2**^	67.83	67.61	67.53	66.41	66.73	66.09	65.65	64.5	63.61	63.12	61.54
**23-24.9 kg/m**^**2**^	9.85	8.81	9.39	8.93	9.09	9.29	9.38	9.72	10.08	11.04	11.77
**≥25 kg/m**^**2**^	3.26	3.14	3.47	3.44	3.71	4.00	4.07	5.15	5.44	6.84	7.82
**Net gestational weght gain(Kg, mean ± SD)#**	7.80 ± 4.80	8.75 ± 4.81	8.66 ± 4.69	9.35 ± 4.67	9.66 ± 4.65	10.13 ± 4.63	10.22 ± 4.59	10.11 ± 4.86	10.32 ± 5.01	10.05 ± 5.28	10.09 ± 5.44
Net gestational weight gain (%)*											
**< 6.5 kg**	38.44	31.13	31.55	25.83	23.38	20.43	19.43	20.62	19.82	21.41	21.59
**6.5~12.5 kg**	46.04	48.34	48.54	50.52	50.57	50.45	50.57	49.19	47.47	46.87	45.28
**> 12.5 kg**	15.52	20.53	19.91	23.65	26.06	29.12	30.00	30.2	32.71	31.72	33.13
City (%)*	21.87	24.86	23.95	29.36	31.31	32.72	35.36	33.4	36.21	38.27	40.84

Missing data on: maternal age, 0.05%; parity, 0.13%; maternal education, 0.48%; maternal height, 4.36%; maternal early pregnancy BMI, 8.94%; gestational weight gain, 11.00%.

* Based on χ2 test for linear trend

# Based on linear-contrast one-way analysis of variance.

To investigate the association between macrosomia and the potential explanatory variables among infants born at ≥37 weeks of gestation, we computed unadjusted and adjusted ORs with 95% confidence intervals (Table 2). The maternal factors most strongly associated with macrosomia were early pregnancy BMI and net gestational weight gain. Compared with women with normal weight (BMI 18.5-22.9), overweight (BMI 23-24.9) and obese (BMI≥25) women, respectively, had a nearly 2-fold and a more than 3-fold risks of delivering a neonatal macrosomia. Net gestational weight gain > 12.5 kg was associated with less than 2-fold increased odds of having a macrosomia birth compared with net gestational weight gain of 6.5-12.5 kg. The possibility of having a macrosomic infant also increased with parity, maternal age, height, level of education, male infants and living in a city. Compared with birth at 40 weeks, gestational age greater than 40 weeks was also associated with an increased risk of delivering a macrosomic infant.

**Table 2:** Crude and adjusted odds ratio for macrosomia among singleton live births at ≥37 weeks of gestation

	No. births	Macrosomia (%)	Crude OR (95% CI)	Adjusted OR (95% CI)
**Maternal age at delivery(y)**				
**≤24**	293, 199	6.26	0.78(0.76-0.80)	0.86(0.84-0.88)
**25-29**	226, 064	7.90	1.00	1.00
**30-34**	65, 066	10.84	1.42(138-1.46)	1.19(1.15-1.24)
**≥35**	9, 840	11.19	1.47(1.38-1.57)	1.17(1.09-1.25)
**Maternal education**				
**College or above**	47, 738	8.58	1.21(1.16-1.26)	1.26(1.21-1.32)
**High school**	128, 739	7.78	1.08(1.05-1.12)	1.21(1.16-1.25)
**Junior school**	336, 372	7.25	1.01(0.98-1.04)	1.12(1.09-1.16)
**Elementary school or less**	78, 751	7.23	1.00	1.00
**Gestational age(wk)**				
**37**	31, 142	2.40	0.25(0.23-0.27)	0.24(0.22-0.25)
**38**	85, 294	3.77	0.40(0.38-0.42)	0.37(0.36-0.39)
**39**	176, 667	6.17	0.67(0.66-0.69)	0.64(0.63-0.66)
**40**	182, 353	8.93	1.00	1.00
**41**	87, 709	11.20	1.29(1.25-1.32)	1.33(1.30-1.37)
**≥42**	31, 307	10.95	1.26(1.21-1.30)	1.35(1.30-1.41)
**Infant gender**				
**Female**	285, 406	5.38	1.00	1.00
**Male**	309, 066	9.39	1.82(1.79-1.86)	1.95(1.91-1.99)
**Maternal height(cm)**				
**≤156**	146, 228	4.52	0.62(0.61-0.64)	0.65(0.63-0.67)
**157-161**	253, 852	7.03	1.00	1.00
**≥162**	168, 450	10.69	1.57(1.54-1.61)	1.56(1.52-1.59)
**Maternal BMI(kg/m^2^)**				
**< 18.5**	109, 810	3.89	0.52(0.50-0.53)	0.47(0.46-0.49)
**18.5-22.9**	354, 411	7.31	1.00	1.00
**23-24.9**	52, 635	12.09	1.75(1.70-1.80)	1.92(1.87-1.98)
**≥25**	24, 455	16.71	2.56(2.47-2.65)	3.04(2.92-3.16)
**Net gestational weight gain (kg)**				
**< 6.5**	134, 078	5.44	0.77(0.75-0.79)	0.62(0.60-0.64)
**6.5-12.5**	256, 502	6.82	1.00	1.00
**> 12.5**	138, 515	10.66	1.60(157-1.63)	1.72(1.68-1.76)
**Parity**				
**0**	508, 436	6.90	1.00	1.00
**1**	80, 236	10.78	1.63(1.59-1.67)	1.51(1.46-1.56)
**≥2**	5, 056	11.75	1.80(1.65-1.96)	1.70(1.55-1.87)
**Maternal residence**				
**City**	184, 341	8.36	1.20(1.18-1.23)	1.08(1.06-1.11)
**country**	410, 131	7.07	1.00	1.00

Since the incidence of macrosomia is affected by the duration of gestation, we also examined the trend of LGA, which better reflects fetal growth. We further separated the whole data into two periods: 1994-2000 and 2002-2005, because these two periods had different velocities of weight increase. The crude and adjusted odds ratios associated with yearly changes in LGA are showed in Table 3. In the sequentially adjustment model, adjustment for gender, parity and gestational age enlarged the yearly effect between 1994 and 2000. Major reductions from the crude odds ratios were associated with increase in net gestational weight gain. Between 2002 and 2005, the increasing maternal early pregnancy BMI changed the observed trend. After simultaneously adjusting for those potential factors, the trend of LGA declined in recent years.

**Table 3:** Multiple analysis with sequential adjustment for secular trend in LGA between 1994 and 2000 and between 2002 and 2005 among term singleton live births

Yearly effect	Number	Odds ratio(95% confidence intervals)
**1994-2000**		
**Unadjusted(Crude)**	380, 184	1.056(1.051-1.061)
**Adjusted for Gender**	380, 184	1.057(1.052-1.062)
+ **Parity**	379, 464	1.059(1.055-1.064)
+ **Gestational age**	379, 464	1.060(1.056-1.065)
+ **Maternal age**	379, 208	1.056(1.051-1.061)
+ **Maternal Height**	356, 945	1.054(1.049-1.059)
+**Maternal early pregnancy BMI**	334, 344	1.053(1.048-1.058)
+ **Net gestational weight gain**	328, 077	1.033(1.028-1.037)
+ **Maternal education**	326, 992	1.029(1.024-1.034)
**+ Maternal residence**	326, 992	1.029(1.024-1.033)
**2002-2005**		
**Unadjusted(Crude)**	182, 981	0.996(0.986-1.007)
**Adjusted for Gender**	182, 981	0.997(0.986-1.007)
+ **Parity**	182, 979	0.995(0.985-1.006)
+ **Gestational age**	182, 979	0.993(0.982-1.004)
+ **Maternal age**	182, 965	0.989(0.978-0.999)
+ **Maternal Height**	181, 860	0.986(0.975-0.996)
+**Maternal early pregnancy BMI**	179, 718	0.973(0.962-0.983)
+ **Net gestational weight gain**	173, 996	0.970(0.959-0.980)
+ **Maternal education**	172, 983	0.966(0.956-0.977)
**+ Maternal residence**	172, 983	0.965(0.954-0.976)

## Discussion

Our population-based data show that the mean birth weight, proportion of macrosomia and LGA increased overall in southeastern China from 1994 to 2005. The most important risk factor for the increase in macrosomia rate is net gestational weight gain. The plateau of macrosomia rate in recent years may be caused by declining fetal growth as a result of efforts to control net gestational weight gain as well as earlier delivery. This observation was consistent with findings from a hospital-based study from 1981 to 2006 [25].

Changes in mean birth weight and incidence of macrosomia appear to have gone through two distinct periods: a rapid rise from 1994 to 2000 and a relative plateau from 2002 to 2005. This is parallel to the prelabor caesarean delivery rate: between 1994 and 2000 the rate of prelabor caesarean delivery rose from 20% in 1994 to 40% in 2000, and to 53% by 2002. It remained relatively stable from 2002 through 2005. The jump in rate of prelabor caesarean delivery was associated with a decrease in gestational age, and more macrosomic infants delivered by caesarean delivery. The increase in prelabor caesarean delivery may have been due to concerns about related complications for larger infants prompting delivery at an earlier gestational age. This finding corroborates observations reported by Schack-Nielsen [7], using Danish national data and by Wills from Queensland, Australia [26]. The decrease in gestational age to some degree offset the effects of rising gestational weight gain, maternal BMI and other changes in maternal and infants characteristics. This finding is also consistent with an ecological study by Zhang et al showing that a rising rate of labor induction in the US population was significantly associated with reduced birth weight, gestational age and rate of macrosomia [14].

The effect of gestational weight gain on birth weight may differ according to maternal prepregnant BMI. Most studies suggest that pregnancy weight gain influences birth weight more in women who are underweight than in women who are overweight [27,28]. Chinese women still have a high proportion of normal prepregnancy BMI. This could possibly explain why change in net gestational weight gain rather than prepregnancy BMI contributed more to the increase in LGA in Chinese population.

The changing trends of macrosomia rate were associated with the dietary structure. In the 1990's, with economic development and improvement of living standard, nutrition and diet during pregnancy also improved. The dietary structure in Chinese population changed towards high calorie, high fat and low fibre. Women gained more weight and fetus grew faster. Net gestational weight gain reached its peak in 2000. This was temporally related to a concerted effort begun in 2001 to have the mother gain only an appropriate amount of weight during pregnancy. From 2002 through 2005 the net gestational weight gain plateaued, and so did the rate of macrosomic infants.

The strength of our study was the use of surveillance data that provides a large population size to allow examination of small yet consistent changes in macrosomia over time. Unlike smaller and hospital-base studies, the population-based study permits us to generalize the findings to substantially larger areas of China. Second, our study area is one of the most developed regions in China. This is reflected in the great improvement in women's education. Thanks to economic reforms China has undertaken over the past few decades, many regions have substantially improved living conditions. Therefore, the trends observed in our study are instructive for the rest of China.

Limitations of our data should also be noted. Our surveillance data did not record gestational age based on ultrasound dating. Gestational age based on the first date of last menstrual period has errors particularly among preterm and postterm births [29]. Second, we have no reliable information on pre-existing or gestational diabetes. Finally, reduction of maternal smoking during pregnancy is an important factor for macrosomia increase in developed countries [6,8,10,11]. Although we did not have information on maternal smoking during pregnancy, the proportion of smokers among Chinese women was very small (3.8%)[30], and pregnant women generally avoided such risk behaviour. Therefore, smoking during pregnancy is not a major issue in our population.

## Conclusions

In southeast China, the incidence of macrosomia increased from 1994 to 2000 and was mainly related to increasing net gestation weight gain. The incidence of macrosomia has levelled off in recent years partly due to increasing use of prelabor caesarean delivery and earlier delivery and partly due to moderation of gestational weight gain.

## Competing interests

The authors declare that they have no competing interests.

## Authors' contributions

YL, JZ, and ZL were responsible for the conception, design and acquisition of data. YL was responsible for the analysis and interpretation of data and drafting the initial manuscript. JZ revised it critically for important intellectual content. XL and WX helped for the data analysis and modification. ZL were responsible for reviewing all drafts of the manuscript and giving final approval of the version to be published. All authors read and approved the final manuscript.

## Pre-publication history

The pre-publication history for this paper can be accessed here:

http://www.biomedcentral.com/1471-2458/11/818/prepub
